# Treatment of malignant pleural mesothelioma by fibroblast activation protein-specific re-directed T cells

**DOI:** 10.1186/1479-5876-11-187

**Published:** 2013-08-12

**Authors:** Petra C Schuberth, Christian Hagedorn, Shawn M Jensen, Pratiksha Gulati, Maries van den Broek, Axel Mischo, Alex Soltermann, Astrid Jüngel, Osiris Marroquin Belaunzaran, Rolf Stahel, Christoph Renner, Ulf Petrausch

**Affiliations:** 1 Department of Oncology, University Hospital Zurich, Rämistr. 100, 8091 Zürich, Switzerland; 2 Laboratory of Molecular and Tumor Immunology, Earle A. Chiles Research Institute, Providence Cancer Center and Providence Portland Medical Center, 4805 NE Glisan St., Portland, OR 97213, USA; 3 Institute of Surgical Pathology, University Hospital Zurich, Schmelzbergstr.12, 8091 Zurich, Switzerland; 4 Center of Experimental Rheumatology, University Hospital Zurich, Gloriastr. 23, 8091 Zürich, Switzerland; 5 Department of Immunology, University Hospital Zurich, Rämistr. 100, 8091 Zürich, Switzerland

## Abstract

**Introduction:**

Malignant pleural mesothelioma (MPM) is an incurable malignant disease, which results from chronic exposition to asbestos in at least 70% of the cases. Fibroblast activation protein (FAP) is predominantly expressed on the surface of reactive tumor-associated fibroblasts as well as on particular cancer types. Because of its expression on the cell surface, FAP is an attractive target for adoptive T cell therapy. T cells can be re-directed by retroviral transfer of chimeric antigen receptors (CAR) against tumor-associated antigens (TAA) and therefore represent a therapeutic strategy of adoptive immunotherapy.

**Methods:**

To evaluate FAP expression immunohistochemistry was performed in tumor tissue from MPM patients. CD8^+^ human T cells were retrovirally transduced with an anti-FAP-F19-∆CD28/CD3ζ-CAR. T cell function was evaluated *in vitro* by cytokine release and cytotoxicity assays. *In vivo* function was tested with an intraperitoneal xenograft tumor model in immunodeficient mice.

**Results:**

FAP was found to be expressed in all subtypes of MPM. Additionally, FAP expression was evaluated in healthy adult tissue samples and was only detected in specific areas in the pancreas, the placenta and very weakly for cervix and uterus. Expression of the anti-FAP-F19-∆CD28/CD3ζ-CAR in CD8^+^ T cells resulted in antigen-specific IFNγ release. Additionally, FAP-specific re-directed T cells lysed FAP positive mesothelioma cells and inflammatory fibroblasts in an antigen-specific manner *in vitro*. Furthermore, FAP-specific re-directed T cells inhibited the growth of FAP positive human tumor cells in the peritoneal cavity of mice and significantly prolonged survival of mice.

**Conclusion:**

FAP re-directed CD8^+^ T cells showed antigen-specific functionality *in vitro* and *in vivo*. Furthermore, FAP expression was verified in all MPM histotypes. Therefore, our data support performing a phase I clinical trial in which MPM patients are treated with adoptively transferred FAP-specific re-directed T cells.

## Introduction

Malignant pleural mesothelioma (MPM) is a rare solid organ tumor, which originates from malignant transformed cells of the mesothelium [1]. A causal link to environmental factors has been established as at least 70% of MPM patients have a definite record of chronic asbestos exposure [2]. MPM is considered an incurable disease with a median survival of 2 years even when intensive multi-modality treatment is performed [3-5]. Therefore, new therapeutic options are desperately needed.

Adoptive transfer of modified (re-directed) autologous T cells is a promising therapeutic strategy and objective responses were observed in preliminary clinical trials [6]. Re-directed T cells are (autologous) T cells that are retrovirally transduced to express a chimeric antigen receptor (CAR) specific for a target on the cell surface. CARs contain a single-chain Fv (scFv)-based molecule, which is specific for the target and coupled to T cell-specific signaling moieties such as CD28 and CD3ζ. The general functionality of these CARs is documented widely by recent literature [7]. The most striking success was observed in B cell lymphoma patients when CD19 was used as an immunological target [8]. However, also in solid cancer types such as ovarian cancer [9] and neuroblastoma [10] adoptive T cell transfer was tested. Recently, we successfully generated peptide-specific re-directed T cells targeting NY-ESO-1 [11]. We tested different signaling domains of the chimeric antigen receptors (CARs) and could observe *in vivo* and *in vitro* immunological functionality [12].

To treat MPM with re-directed T cells, we set out to identify a surface protein that is universally expressed by the majority of MPM subtypes (epithelioid, sarcomatoid and biphasic). Fibroblast activation protein (FAP) was suggested to be a potential target antigen since FAP is widely expressed by various epithelial and mesenchymal cancer types [13]. FAP expression has been studied extensively by immunohistochemistry in the past [14] and is known to differ between cell types and even within the tumor tissue. Two patterns of expression are most frequently found: 1) FAP expression by cancer associated fibroblasts (CAFs) of the tumor stroma only (e.g. breast or colorectal cancer [15]) or 2) by both the tumor stroma and the tumor cells (e.g. sarcoma [16]). Altogether, FAP is expressed in about 90% of most common cancer types like breast, lung and colorectal cancer [17]. Its expression is also associated with chronic inflammation, tissue remodeling [18] and immune modulation in the tumor tissue [19]. We show here that FAP is expressed in all three major MPM histotypes, namely the epithelioid, sarcomatoid and the intermediate called biphasic.

FAP has been validated as target antigen in oncology by a monoclonal antibody called F19 (humanized version: sibrotuzumab) in different phase I/II clinical trials [20,21]. The antibody recognizes exclusively non-degraded human FAP. F19 accumulated specifically in the tumor tissue [22]; however, the clinical effect was marginal. The results indicated that the sole use of an antibody was not sufficient to induce a meaningful immunological anti-tumor response. Therefore, F19 was not further developed for clinical use [21].

We developed re-directed T cells with a CAR consisting of a scFv of the FAP-specific F19 antibody, a CD28 signaling domain lacking the lck binding moiety [23] and a CD3ζ signaling domain. Our rational to develop FAP-specific re-directed T cells based on the F19 antibody was to utilize its already clinically proven specificity to target FAP positive tumor tissue combined with the immunological effector function of T cells. As observed by others our previous results clearly indicated increased antigen-specific function of re-directed T cells when the CAR contained a CD28 signaling domain [12,24]. Therefore, we decided to generate a second generation CAR with a co-stimulating signal provided by the CD28 domain. For the first time we show here that re-directed T cells specific for FAP are cytotoxic towards FAP positive targets *in vitro* and control xenografted human FAP positive tumors *in vivo*.

## Material and methods

### Cell lines

293T and MSTO-211H were purchased from ATCC (Manassas, VA). HT1080FAP are HT1080 cells stably transfected with human FAP and HT1080PA are mock-transfected HT1080 cells [25]. T2-1B cells are HLA-A*02:01-positive T2 cells stably transfected with the HLA-A*02:01-restricted NY-ESO-1 peptide 157–165 [26]. Tumor cell lines were cultivated in standard R10 media (RPMI1640 GlutaMax supplemented with 10% fetal bovine serum (FBS) (v/v), 50 U/ml penicillin and 50 μg/ml streptomycin; all obtained from Invitrogen (Karlsruhe, Germany)). For the culture of transfected HT1080 and T2 cells, 200 μg/ml G418 (Sigma-Aldrich Chemie GmbH, Buchs, Switzerland) or 2.5 μg/ml Hygromycin B (Invitrogen, Karlsruhe, Germany) was added, respectively.

To enable *in vivo* imaging, HT1080FAP and HT1080PA cells were stably transfected with a D-firefly luciferase encoding plasmid (pGL4.26 plasmid, Promega, Dübendorf, Switzerland which was kindly provided by Martin Pruschy, University Hospital Zurich, Switzerland) using Fugene transfection reagent (Roche Diagnostics GmbH, Mannheim, Germany) according to the manufacturer’s protocol. Forty-eight hours after transfection cells were submitted to selection using 150 μg/ml Hygromycin B. Cells were cloned by limited dilution and luciferase expression was monitored using Bright-Glo™ Luciferase Assay System and a GloMax Microplate Luminometer (both Promega, Madison, WI) according to the manufacturer’s protocol. Clones with luciferase activity underwent two more rounds of limited dilution followed by further testing for luciferase activity. Finally, a stable HT1080FAP-luc and HT1080PA-luc clone were selected that exhibited high luciferase activity and maintained this over months even when cultured in the absence of Hygromycin B. The resultant HT1080FAP-luc and HT1080PA-luc cells were cultivated in standard R10 media supplemented with 200 μg/ml G418 and 150 μg/ml Hygromycin B.

Rheumatoid arthritis synovial fibroblasts originating from tissues obtained during joint replacement surgery (Schulthess Clinic, Zurich, Switzerland) were isolated for cell cultures as described previously [27]. Briefly, synovial tissues were digested with dispase at 37°C for 60 minutes. After washing, cells were grown in Dulbecco’s MEM NUT MIX-F12 (Invitrogen, Karlsruhe, Germany) supplemented with 10% FBS, 50 U/ml penicillin, 50 μg/ml streptomycin and 10 mM HEPES (Invitrogen, Karlsruhe, Germany).

### Antibodies and reagents

Antibodies for flow cytometry were purchased from eBioscience (San Diego, CA) (anti-human CD8a-FITC), Invitrogen (Karlsruhe, Germany) (LIVE⁄DEAD Fixable Aqua Dead Cell Stain Kit) and Southern Biotech (Birmingham, AL) (anti-human IgG-PE). Analysis of FAP expression was performed using the humanized anti-F19 antibody [20] (kindly provided by Andrew Scott, Ludwig Institute for Cancer Research, Australia), whereas MabThera (Rituximab; anti CD20 mAb; Roche Pharma AG, Reinach, Switzerland) was used as a negative control antibody. Both antibodies were directly labeled with Alexa Fluor 647 (Alexa Fluor^®^ 647 Antibody Labeling Kit; Invitrogen, Karlsruhe, Germany) according to the manufacturer’s protocol. Murine F19 antibody [25] (kindly provided by Andrew Scott, Ludwig Institute for Cancer Research, Australia) was used as primary antibody for immunohistochemistry staining, and the murine HD6 monoclonal antibody served as a control antibody [28]. A biotin-labeled goat anti mouse IgG (Fcγ fragment specific) was used as secondary antibody (Jackson ImmunoResearch, Suffolk, UK). Recombinant human FAP was kindly provided by Andrew Scott, Ludwig Institute for Cancer Research, Australia.

### Generation of the FAP-specific chimeric antigen receptor (CAR)

The variant heavy and the variant light chain of humanized F19 [21] have been converted into a scFv fragment and were flanked with NcoI and BamHI restriction sites. This construct was cloned into the pBullet vector [29] containing a human ∆CH2/CH3 domain, a ∆CD28 [23] and a CD3ζ signaling domain (kindly provided by Hinrich Abken, University of Cologne, Germany). The human CH2/CH3 domain has been modified to reduce FcγR binding and thereby minimizing the risk of off-target T cell activation by CAR binding to FcγR^+^ cells [30]. Furthermore, the CD28 binding domain for lck was corrupted by site directed mutagenesis to avoid IL-2 release and subsequent persistence of T_reg_ cells [23].

The resulting chimeric antigen receptor construct was termed anti-FAP-F19-∆CD28/CD3ζ. Anti-NY-ESO-1-T1-∆CD28/CD3ζ recognizing the HLA-A*02:01/ NY-ESO-1_157-165_ peptide complex served as a control construct [12]. It displays the same genetic modifications in the downstream CH2/CH3 and CD28 lck domains.

### Retroviral transduction of peripheral blood CD8^+^ T cells

Retroviral transduction of human peripheral CD8^+^ T cells was performed as previously described [12] with following minor modifications. CD8^+^ T cells were purified from healthy donor buffy coats using anti-CD8 labeled magnetic beads and MACS technology (Miltenyi, Bergisch Gladbach, Germany). Positive selection with anti-human CD8 microbeads typically resulted in a ≥ 95% pure CD8^+^ population. CD8^+^ T cells were subsequently activated with CD3/CD28 human T cell expander beads (Invitrogen, Germany) at a bead to cell ratio of 1:5 and 100 IU/ml IL-2 (ImmunoTools, Friesoythe, Germany) in standard R10 media for 48 h. Activated CD8^+^ T cells were retrovirally transduced for 48 hours in the presence of 100 IU/ml IL-2 by co-cultivation with 293T cells that were transiently producing high titers of infectious retrovirus carrying the genomic information of the chimeric antigen receptors. CD8^+^ T cells were expanded for 2 more days in R10 plus 100 IU/ml IL-2 before harvest and subsequent application in experiments.

### Flow cytometry

Flow cytometry was carried out as described previously [12]. Live/dead staining was performed using the LIVE/DEAD fixable Aqua Dead Cell Stain Kit according to the manufacturer’s protocol. BrdU Proliferation assay were performed with the FITC BrdU flow kit (BD Pharmingen, San Diego, CA) according to the manufacturer’s instruction. Flow cytometric measurements were performed using a FACSCanto II or FACSCalibur machine (BD Biosciences, San Diego, CA). Data were analyzed using FlowJo software (Tree Star, Ashland, OR).

### Cytokine assays

Cytokine production was assessed by sandwich ELISA assays. Supernatants of co-cultivated effector and target cells were collected after 24 hours of incubation. IFNγ and IL-2 levels were detected using BD OptEIA set human IFNγ and BD OptEIA set human IL-2 kits, respectively, according to the manufacturer’s instruction (BD Biosciences, San Diego, CA).

### Immunohistochemistry

To study potential off-target sites of FAP re-directed T cells, we purchased frozen tissue arrays that contained human adult normal tissue (BioChain, Newark, CA) and stained them for FAP expression. To study expression of FAP in MPM, a total of 9 fresh-frozen samples from malignant pleural mesothelioma patients were retrieved from the biobank of the Institute of Surgical Pathology, University Hospital Zurich.

To perform immunohistochemistry acetone-fixed tissue slides were developed employing the Vectastain ABC Kit Peroxidase Mouse IgG (VECTOR LABORATORIES, Burlingame, CA) according to the manufacturer’s protocol with minor modifications. Briefly, slides were additionally incubated with an avidin and a biotin block (both VECTOR LABORATORIES, Burlingame, CA) for 10 min at RT. Two μg/ml murine F19 or murine HD6 antibody as negative control in 20% of blocking buffer (10% rabbit serum (VECTOR LABORATORIES, Burlingame, CA), 5% milk powder (Roth, Karlsruhe, Germany) in PBS) were added as primary antibody overnight at 4°C. The secondary biotin-goat anti mouse Fc antibody was diluted 1:2000 in 20% of blocking buffer. ImmPACT DAB, SK-4105 (VECTOR LABORATORIES, Burlingame, CA) was used as peroxidase substrate kit according to the manufacturer’s protocol.

The slides were analyzed with a high-speed wide field fluorescence microscope Leica LX and pictures were recorded using a Leica DFC 350 FX camera system (both Leica Microsystems, Heerbrugg, Switzerland).

### Europium release assay

Specific cytotoxicity of re-directed T cells was analyzed by a europium release assay (Perkin Elmer, Waltham, MA) as previously described [12]. HT1080FAP-luc, HT1080PA-luc, MSTO-211H, T2-1B and primary rheumatoid fibroblasts served as target cells for anti-FAP-F19-∆CD28/CD3ζ and anti-NY-ESO-1-T1-∆CD28/CD3ζ re-directed T cells. All cell lines were labeled with 2,2’:6’,2”-terpyridine-6,6”- dicarboxylic acid acetoxymethylester (BATDA) for 30 min at 37°C. Target cells were seeded at a density of 10^4^ cells per 96-round bottom-well and co-cultured with effector cells at different effector:target ratios for 4 h at 37°C in R10 media followed by supernatant analyses in a time-resolved Victor^2^ flourometer (Perkin Elmer, Waltham, MA). The specific cytolysis of target cells (%) was calculated by the equation: 100x[experimental release(counts) - spontaneous release(counts)]/[maximum release(counts) - spontaneous release(counts)]

### Therapy of xenografted FAP^+^ human tumors

NOD.Cg-Prkdc^scid^ Il2rg^tm1Wjl^/SzJ mice, commonly known as NOD.scid.γcKO (NSG), were originally obtained from Jackson Laboratories (Bar Harbor, ME, USA), bred and maintained at the University Hospital Zurich under specific pathogen-free conditions. HT1080FAP-luc tumor cells (10^6^ cells per mouse) were co-injected intraperitoneally with re-directed T cells at an effector to target ratio of 5:1. Tumor burden was monitored by body weight measurements and *in vivo* bioluminescence imaging. For that purpose, mice were anesthetized with 2% isoflurane and i.p. injected with 150 mg/kg D-Luciferin (Caliper Life Sciences, Hopkinton, MA) and signals were visualized using IVIS^®^200 Caliper (Caliper Life Sciences, Hopkinton, MA). Monitoring of mice was started immediately after D-Luciferin injection and lasted up to 20 min to obtain the peak photon emission of each animal. Bioluminescence signals were collected and converted to photons/second/cm^2^/steradian in order to normalize each setting for F-stop, exposure time, binning and animal size using Living Image 3.2 (Caliper Life Sciences, Hopkinton, MA). A constant region of interest was designated around the torso of each animal in order to avoid any incoherence. For imaging purposes, a pseudocolor map representing light intensity was superimposed over a whole-body image. Age-matched mice were used for all experiments. Tumor-bearing mice were euthanized if their body weight increased or decreased more than 15% if compared to the weight of non-diseased, age-matched mice or if mice were confined in their normal behavior by tumor growth. Animal experiments were performed according to Swiss federal and cantonal laws on animal protection.

### Statistical analysis

Data were analyzed with Graph Pad Prism version 5.00 for Windows (GraphPad Software, San Diego, CA). Student’s unpaired 2-tailed t-tests were performed between two groups of interest. Survival analysis was performed employing Kaplan Meier survival curves and significance between two variables was calculated using the log-rank test.

## Results

### Malignant pleural mesothelioma expressed fibroblast activation protein in the tumor-associated stroma and on tumor cells

We analyzed the three distinct histologic subtypes of malignant pleural mesothelioma from a total of 18 patient biopsies for FAP expression. The MPM histosubtype was determined by a panel of positive and negative immunohistochemistry (IHC) markers (Calretinin, CK5/6 und TTF1) and morphology. Different cell types in the biopsies were identified by morphology. Staining for FAP was scored by the reference pathologist of our institution (A.S.) in a three-tiered system (+: faint, ++ moderate, and +++ strong).

**Figure 1:**
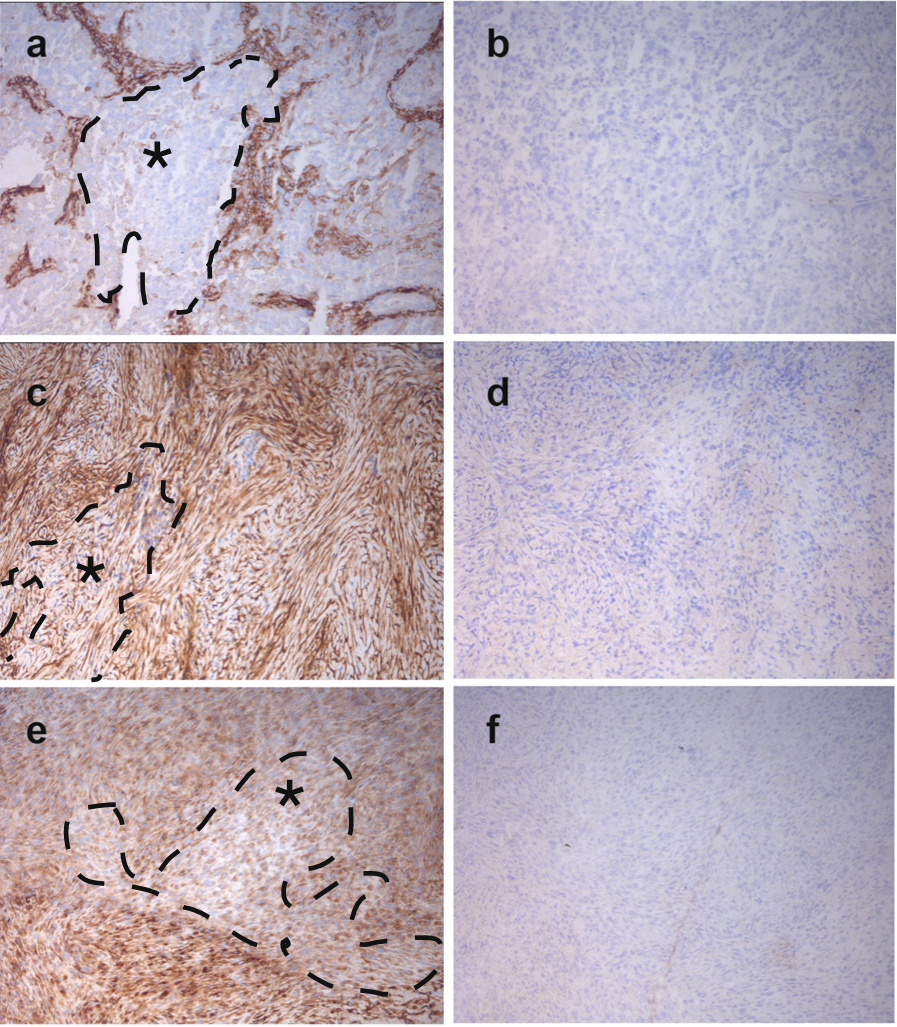
**IHC of fresh-frozen malignant pleural mesothelioma patient sections. A**, **c** and **e** were stained with the FAP recognizing murine F19 antibody, whereas **b**, **d**, **f** represent the secondary system only. All three histosubtypes of MPM have been analyzed: epithelioid **(a**, **b)**, sarcomatoid **(c**, **d)** and bi-phasic **(e**, **f)**. Regions marked with * indicate areas of predominant tumor cell location.

Eight out of eight epithelioid MPM specimens showed faint to strong FAP expression on tumor cells and a moderate to strong expression on stromal cells (Figures 1a,b). Two out for four sarcomatoid MPM samples displayed moderate membranous expression of FAP on tumor cells as well as on stromal cells (Figures 1c,d). All six biphasic MPM samples showed moderate to strong FAP expression on the tumor cells and a strong expression in the stromal compartment (Figures 1e,f). In total, we discovered FAP expression on tumor cells and stroma in 16 out of 18 patient MPM samples.

To evaluate FAP expression in healthy adult tissue we screened 20 organ specimens for FAP expression to determine possible targets of toxic side-effects (adrenal, ovary, pancreas, thyroid, uterus, cervix, breast, placenta, cerebrum, cerebellum, lung, spleen, heart, skin, skeletal muscle, kidney, stomach, small intestine, liver and salivary gland). Specific FAP expression was only present in specific areas of the pancreas and placenta and very weakly for cervix, and uterus (Additional file 1: Figure S1).

### Generation of FAP-specific, re-directed CD8^+^ T cells

We generated re-directed T cells expressing a CAR consisting of a single-chain Fv (scFv) molecule recognizing FAP. The scFv is linked to a modified human CH2/3 immunoglobulin domain to reduce FcRγ binding and a ∆CD28 co-stimulatory domain which lacks the lck binding moiety. The last moiety of the construct is the CD3ζ domain which facilitates intracellular signaling (anti-FAP-F19-∆CD28/CD3ζ). As a control construct we cloned the scFv of the recently published anti-NY-ESO-1-T1-CD28/CD3ζ in the ∆ backbone which in the following is termed anti-NY-ESO-1-T1-∆CD28/CD3ζ [12]. A schematic representation of the CAR re-directed T cells is given in Additional file 1: Figure S2.

**Figure 2:**
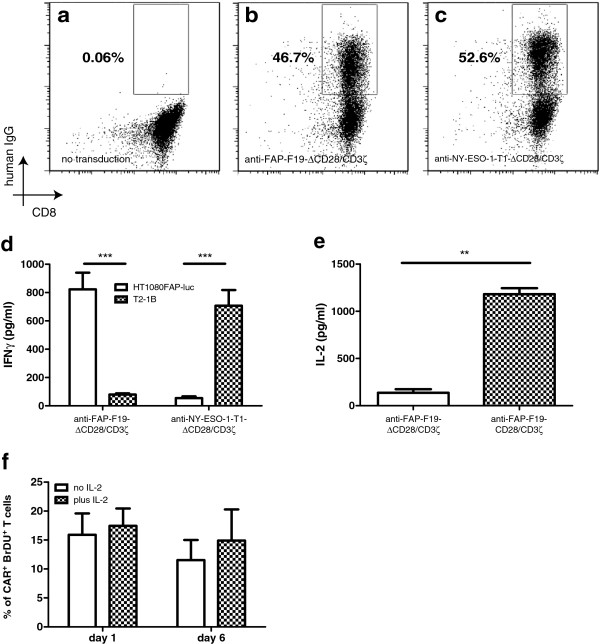
**Transduction efficacy and purity of CD8**^**+ **^**T cells (a-c) and functional activity of CAR transduced T cells *in vitro *(d, e, f).** Transduction efficacy was assessed by staining the CH2/3 immunoglobulin linker domain with anti-hIgG mAb and with anti-CD8 mAb (n = 10). **A** shows non-transduced T cells, whereas **b** displays T cells transduced with the FAP-specific anti-FAP-F19-∆CD28/CD3ζ CAR and **c** with the NY-ESO-1 specific anti-NY-ESO-1-T1-∆CD28/CD3ζ CAR. 24 h co-cultivation of re-directed T cells with the target cell lines HT1080FAP-luc and T2-1B was analyzed for IFNγ release (n = 5) **(d)**. Comparison of IL-2 production of anti-FAP-F19-∆CD28/CD3ζ and anti-FAP-F19-CD28/CD3ζ after 24 h of co-cultivation with HT1080FAP-luc (n = 2) **(e)**. Proliferation of anti-FAP-F19-∆CD28/CD3ζ re-directed T cells after stimulation with recombinant human FAP (1 μg/ml) in the absence and presence of IL-2 (100 IU/ml) at indicated time points (n = 2) **(f)**. Error bars were calculated as mean + SD (***p < 0.001, **p < 0.01 as calculated by Student’s unpaired 2 tailed t-tests).

We isolated CD8^+^ T cells from human peripheral blood and retrovirally transduced them to achieve cell surface expression of CARs which was assessed by detection of the modified human CH2/3 immunoglobulin domain (hIgG) using flow cytometry. After transduction, 46.7% of anti-FAP-F19-∆CD28/CD3ζ re-directed CD8^+^ T cells stained for hIgG (Figure 2b) and 52.6% of CD8^+^ T cells carried the anti-NY-ESO-1-T1-∆CD28/CD3ζ CAR (Figure 2c). Non-transduced CD8^+^ T cells did not stain for hIgG (Figure 2a). The range of transduction efficiency for anti-FAP-F19-∆CD28/CD3ζ was 31.31 ± 10.69 (SD) and for anti-NY-ESO-1-T1-∆CD28/CD3ζ 32.55 ± 10.31 (SD) (Figure 2b,c). To investigate the functionality of the generated re-directed T cells we co-cultivated effector cells and target cells at a 1:1 ratio. Both constructs showed antigen-specific IFNγ release (Figure 2d). Furthermore, to determine the effect of abrogating the lck-binding domain, we activated CD8^+^ T cells expressing anti-FAP-F19-∆CD28/CD3ζ or anti-FAP-F19-CD28/CD3ζ (intact lck-signaling) with recombinant human FAP. Measurement of IL-2 release demonstrated a significant reduction in IL-2 production in anti-FAP-F19-∆CD28/CD3ζ re-directed T cells. However, anti-FAP-F19-∆CD28/CD3ζ re-directed T cells secreted measurable basal amounts of IL-2 (Figure 2f). Since we observed reduced IL-2 production we investigated the ability of anti-FAP-F19-∆CD28/CD3ζ re-directed T cells to proliferate in the absence of exogenous IL-2. Up to day 6 during antigen-specific stimulation the addition of exogenous IL-2 did not increased the proliferation capacity of anti-FAP-F19-∆CD28/CD3ζ re-directed T cells (Figure 2f). To investigate the longevity of re-directed T cells without cytokine support we analyzed the *in vitro* survival of anti-FAP-F19-∆CD28/CD3ζ re-directed T cells with or without supplementation of 100 IU/ml IL-2 employing live/dead staining of CD8^+^ T cells. The addition of IL-2 led in all cases to sustained survival of 70–98% CAR^+^ as well as CAR^-^ T cells (Figure 2d) whereas no IL-2 supplementation resulted in early loss of CAR^+^ T cells starting around day 3 (Figure 2e). CAR^-^ T cells showed a high percentage of living cells out to day 8. However, between day 8 and 12 all IL-2 deprived CD8 T cells were lost regardless of their transduction state (Figure 2e).

### FAP-specific, re-directed T cells lysed FAP positive target cells

**Figure 3:**
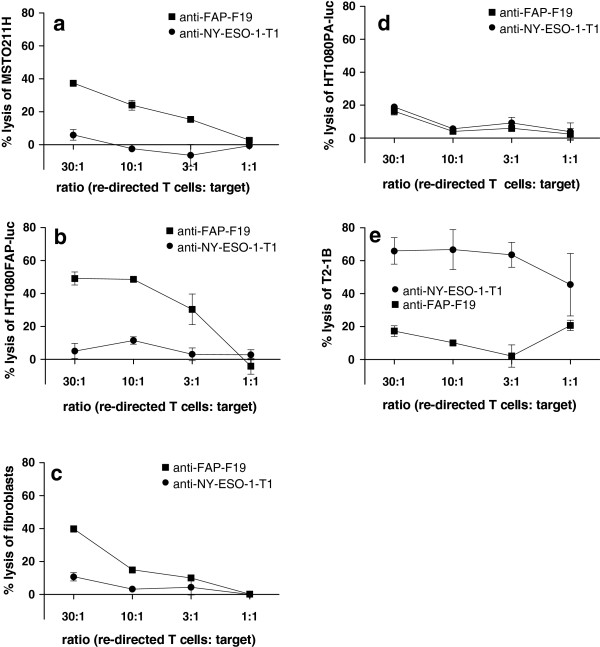
**Antigen-specific cytolysis of anti-FAP-F19-**∆**CD28/CD3**ζ **re-directed T cells.** Re-directed T cells recognizing FAP (anti-FAP-F19-∆CD28/CD3ζ (square) and NY-ESO-1_157-165_ peptide in the HLA-A*02:01 context (anti-NY-ESO-1-T1-∆CD28/CD3ζ) (circle) were cultured with MSTO-211H cells **(a**, FAP positive**)**, HT1080FAP-luc cells **(b**, FAP positive**)**, primary fibroblasts **(c**, FAP positive**)**, HT1080PA-luc cells **(d**, FAP negative**)** and T2-1B cells **(e**, FAP negative but HLA-A*02:01, NY-ESO-1_157-165_ peptide positive**)** at different effector to target ratios and analyzed after 4 h of incubation in a europium release assay (n = 2). Error bars were calculated as mean + SD.

We performed europium cytotoxicity assays to determine antigen-specific cytotoxicity of the re-directed T cells *in vitro*. Therefore, we used different target cells that exhibited various levels of FAP expression. As expected from FAP positive IHC of MPM specimens the mesothelioma derived cell line MSTO-211H showed FAP expression (Additional file 1: Figure S3c). The FAP transduced cell line HT1080FAP-luc (Additional file 1: Figures S3a,b) served as positive control. As expected from previous reports [29] primary fibroblasts from patients with active joint inflammation also stained positive for FAP (Additional file 1: Figure S3e). We employed as control cell lines HT1080PA-luc and T2-1B known to be negative for FAP expression (Additional file 1: Figures S3b,f and g). Importantly, co-cultivation of the mesothelioma cell line MSTO-211H with anti-FAP-F19-∆CD28/CD3ζ re-directed T cells resulted in specific lysis of MSTO-211H cells in a dose dependent manner (Figure 3a). In addition, HT1080FAP and primary fibroblasts were also specifically lysed (Figures 3b,c). Anti-FAP-F19-∆CD28/CD3ζ re-directed T cells exhibited only background lysis when co-cultivated with the FAP negative cell lines HT1080PA-luc and T2-1B (Figures 3d,e). Anti-NY-ESO-1-T1-∆CD28/CD3ζ re-directed T cells did not recognize any target cell lines besides T2-1B cells which express the HLA-A*02:01/NY-ESO-1_157-165_ peptide complex as their specific target structure. These data show that FAP-specific re-directed T cells specifically and efficiently lyse FAP positive targets *in vitro*.

### Adoptive therapy with anti-FAP-F19-**∆**CD28/CD3ζ re-directed T cells resulted in significant delay of FAP^+^ tumor outgrowth in the peritoneum of NSG mice

**Figure 4:**
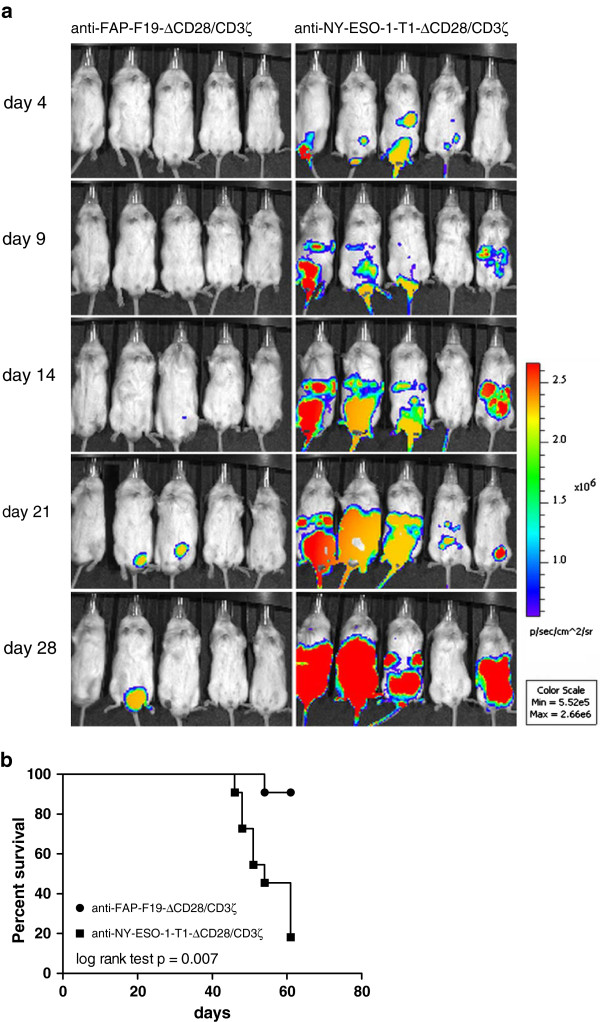
**Xenograft model of disseminated i.p. tumors.** Anti-FAP-F19-∆CD28/CD3ζ or anti-NY-ESO-1-T1-∆CD28/CD3ζ re-directed T cells were co-injected i.p. with 1*10^6^ HT1080FAP-luc tumor cells at an effector to target ratio of 5:1. HT1080FAP-luc tumor cells were recorded by bioluminescence imaging in the same mouse at the indicated days starting day 4 after cell injection **(a)**. A total of 11 mice per experimental setting were treated with re-directed T cells from 2 different donors (donor 1: 6 mice per group, donor 2: 5 mice per group). The median survival of mice was determined by Kaplan-Meier analysis **(b)**. Significance between cohorts was determined by log rank test.

Our findings above demonstrated a FAP-specific re-direction of T cells *in vitro* and a moderate to high FAP expression in MPM samples. The proposed clinical trial is designed to inject re-directed T cells in the pleural space [31]. However, as the pleural space is difficult to assess in mice we established a model in the peritoneum to mimic adoptive transfer of T cells in a body cavity. Therefore, we evaluated the anti-tumor activity of FAP specific re-directed T cells by establishing an i.p. tumor model which constitutively expressed FAP as well as luciferase for *in vivo* detection of tumor cells. Injection of HT1080FAP-luc cells into the peritoneum resulted in progressively growing tumors and mice had to be euthanized within three weeks in accordance to predefined end-points (data not shown). HT1080FAP-luc cells and FAP-re-directed T cells or NY-ESO-1-re-directed T cells were co-injected i.p. and monitored by serial bioluminescent imaging as indicated (Figure 4a). A significant delay and/or protection of tumor outgrowth was detected in the group treated with anti-FAP re-directed T cells, whereas the tumor grew progressively in the control group injected with NY-ESO-1- re-directed T cells. In this allogeneic setting of T cells and tumor cells, NY-ESO-1-specific re-directed T cells displayed no specific anti-tumor effect but prolonged the survival compared to the saline control group (data not shown). Most importantly, there was a significant difference between the anti-FAP and the anti-NY-ESO-1 treated group from day 28 on (P = 0.041, data not shown). In addition, the survival of the experimental groups was monitored. Based on pre-defined end-points in accordance with animal protection mice were euthanized (Figure 4b). The survival after injection of anti-FAP re-directed T cells was significantly longer compared to the control group treated with anti-NY-ESO-1 re-directed T cells (p = 0.007). Median 50% survival was not reached in the group treated with anti-FAP re-directed T cells.

## Discussion

MPM is still a disease in which even the combination of all three conventional branches of oncologic treatment, surgery, chemotherapy and radiotherapy have to be considered a palliative attempt and other therapeutic options have to be evaluated [32]. Beside therapies targeting molecular pathways of the malignant cells [33], immunotherapy is a novel therapeutic strategy to fight MPM [34]. Most of the so far tested approaches focus on the induction of an active-specific immune response. As an alternative approach, passive adoptive immune therapy could be proposed. Therefore, we investigated the potential therapeutic effect of FAP-specific re-directed T cells as a new option for treatment of MPM. All three histological subtypes of MPM demonstrated FAP expression by the tumor stroma and by most tumor cells. Garin-Chesa and colleagues had demonstrated FAP expression in 7 MPM samples in the tumor stroma as well as on tumor cells with variable FAP expression levels [13]. T cells expressing anti-FAP CARs at high level were generated by standard procedures and these re-directed T cells were antigen-specific as characterized by their specific *in vitro* killing of FAP positive mesothelioma cells. To examine the therapeutic efficacy of FAP-specific re-directed T cells *in vivo*, we adoptively transferred re-directed T cells into the peritoneal cavity of mice bearing FAP + tumors. In this setting, FAP-specific re-directed T cells showed strong therapeutic potential, preventing the growth of FAP + tumors and increasing survival of mice.

MPM derives from the mesothelial cell lineage of the serous membranes of the pleura. This process causes a chronic stimulation of the serous membranes resulting in pleural effusion in the majority of clinical cases. Since our first planned clinical protocol (manuscript is published: Petrausch U, Schuberth PC, Hagedorn C, Soltermann A, Tomaszek S, Stahel R, Weder W, Renner C: Re-directed T cells for the treatment of fibroblast activation protein (FAP)-positive malignant pleural mesothelioma (FAPME-1). BMC Cancer 2012, 12:615.) is designed to test the safety of FAP-specific re-directed T cells after injection in the pleural effusion, we developed an intra-peritoneal model for the adoptive transfer of T cells to model the function of these cells in a body cavity. As shown by June and coworkers for mesothelin-specific re-directed T cells [35], the application of FAP-specific re-directed T cells resulted in the specific lysis of the respective target cells. Since the chosen model system tested human CD8 positive cells in a murine xenograft model with human tumor cells, the potential for allogeneic effects is a major concern. To control for this allogeneic effect, we used our previously, extensively characterized HLA-A*02:01/NY-ESO-1_157-165_ peptide-specific re-directed T cells because these cells displayed some allogeneic reactivity [12]. Importantly, HLA-A*02:01/NY-ESO-1_157-165_ peptide-specific re-directed T cells could not control the growth of FAP + tumor cells demonstrating the antigen-specific therapeutic effect of our re-directed FAP-specific T cells.

Adoptive transfer of T cells with a new specificity generated by gene transfer is associated with two major concerns. First, the new specificity can cause on-target off-site effects resulting in T-cell mediated organ damage as documented by recent publications [36]. Therefore, the exploration of off-site target expression is of crucial interest. In healthy tissue, low-level expression of FAP could only be detected in pancreas, placenta, cervix and uterus. We have not tested tissue from chronically inflamed tissue. Chronic inflammation of tissues causes activation of fibroblasts as part of the remodeling process which occurs during inflammation [18]. To estimate the effect of re-directed FAP-specific T cells in chronically inflamed tissue, we used activated fibroblasts from patients with rheumatoid arthritis out of the effusion from inflamed joints. As reported, the herein used activated fibroblasts isolated from different donors expressed FAP [37]. Therefore, the strong and antigen-specific lysis of fibroblasts derived from inflamed tissue by re-directed FAP-specific T cells was expected. This observation indicates the potential for adverse off-site effects in tissues with activated fibroblasts. The F19 CAR cannot be used in murine models of inflammation since the scFv only recognizes the human version of FAP. Since no other model for toxicity estimation is available with the F19 CAR we performed cytotoxicity assay with human fibroblast and implemented strict exclusion criteria for our planed clinical study. All patients with chronic inflammatory diseases have been excluded. Patients with coronary heart disease (CHD), stroke or peripheral vascular disease (PVD) also have to be excluded since expression of FAP was detected in atherosclerotic vessels [31,38,39].

To further minimize potential long-term off-target toxicity we evaluated the therapeutic potential of a second generation CAR with a CD28 co-stimulatory domain. We and other have seen, that re-directed CD8+ T cells with CAR containing a CD28 moiety eliminated target cells more effectively than re-directed T cells with a CD3 domain alone [12,40]. CD28 signaling also induces IL-2 production, which leads to expansion of cells [41]. The herein used CAR lacks the lck domain, avoiding IL-2 induction [23]. The co-stimulation by 4-1BB also enhances persistence and clonal expansion of re-directed T cells, whereas cytotoxic activity is less impacted [42]. Based on the most recent literature we designed a FAP-specific CAR causing cell lysis by CD8+ T cells and minimal long-term persistence. Based on the existing experience this allows a clinical phase I trial to first test a CAR recognizing FAP, for which no clinical data are available so far [6].

The second concern is the gene transfer itself. The clinical course of patients with X-linked immunodeficiency after gene transfer in hematopoietic stem cells resulted in the development of leukemia in 4 out of 9 cases [43]. Most recently, the safety of retroviral gene transfer in T cells in clinical settings was confirmed by measuring 540 patient years of follow up [44]. During this time, retroviral gene transfer resulted in no malignant transformation even in cases with very high numbers of integrations per cell (2x10^11^ integration site per cell). These recent data suggest that T cells can be safely transduced by retroviral transfer. Additionally, our re-directed FAP-specific T cells showed limited *in vitro* survival in the absence of exogenous IL-2 suggesting that retroviral transduction did not result in altered survival control. The limited life span implies a predominant effector phenotype of terminally differentiated T cells as was previously shown for HLA-A*02:01/NY-ESO-1_157-165_ re-directed T cells [12]. These data also suggest that gene transfer did not alter this differentiation program of T cells allowing them to develop in functional effector T cells.

## Conclusion

Taken together, the presented data give a strong rationale to test re-directed FAP-specific T cells in patients with MPM since *in vitro* and *in vivo* functionality was clearly demonstrated and FAP expression is present in all subtypes of MPM. However, special caution is necessary in the selection of patients. Patients with chronic inflammatory diseases should be excluded when targeting FAP + tumors with FAP-specific re-directed T cells. Based on these data, we recommend the adoptive transfer of re-directed T cells in a loco-regional fashion to minimize immediate systemic distribution and unexpected recognition of FAP in off-site tissues. As loco-regional therapy, adoptive transfer could be performed in body cavities like pleura or peritoneum or directly in the tumor tissue [45]. This approach could also lead to a reduction of the number of adoptively transferred cells resulting in small scale production of re-directed T cells with reduced costs and fewer technical hurdles for GMP production. Due to the expression pattern of FAP in malignant tissues FAP specific re-directed T cells have the potential to demonstrate anti-tumor effects besides MPM in a wide variety of different cancers.

## Consent

This study was in accordance with Swiss laws and approved by the ethical committee of the Kanton Zurich (ref. nr. Stv.29–2009 and KEK No. 475).

## Competing interests

The authors declare that they have no competing interests.

## Authors’ contributions

PCS, SMJ, CR, UP designed the research plan. PCS, UP wrote the manuscript. PCS, CH, PG, OMB performed IHC, killing assays, ELISAs, animal experiments. PCS, UP analyzed data, created the figures, performed statistics. AS performed histology reports. AJ, AM, AS, RS, MvB provided vital reagents/materials/animals. All authors read and approved the final manuscript.

## Supplementary Material

Additional file 1 Supplemental figures. Click here for file
