# Supplementary material for: Treatment of malignant pleural mesothelioma by fibroblast activation protein-specific re-directed T cells
Source: J Transl Med. 2013 Aug 12;11:187. doi: 10.1186/1479-5876-11-187 (PMC3751305; doi:10.1186/1479-5876-11-187)
Supplement: Additional file 1 — Supplemental figures. [file 1479-5876-11-187-S1.pdf]

supplemental figure 1

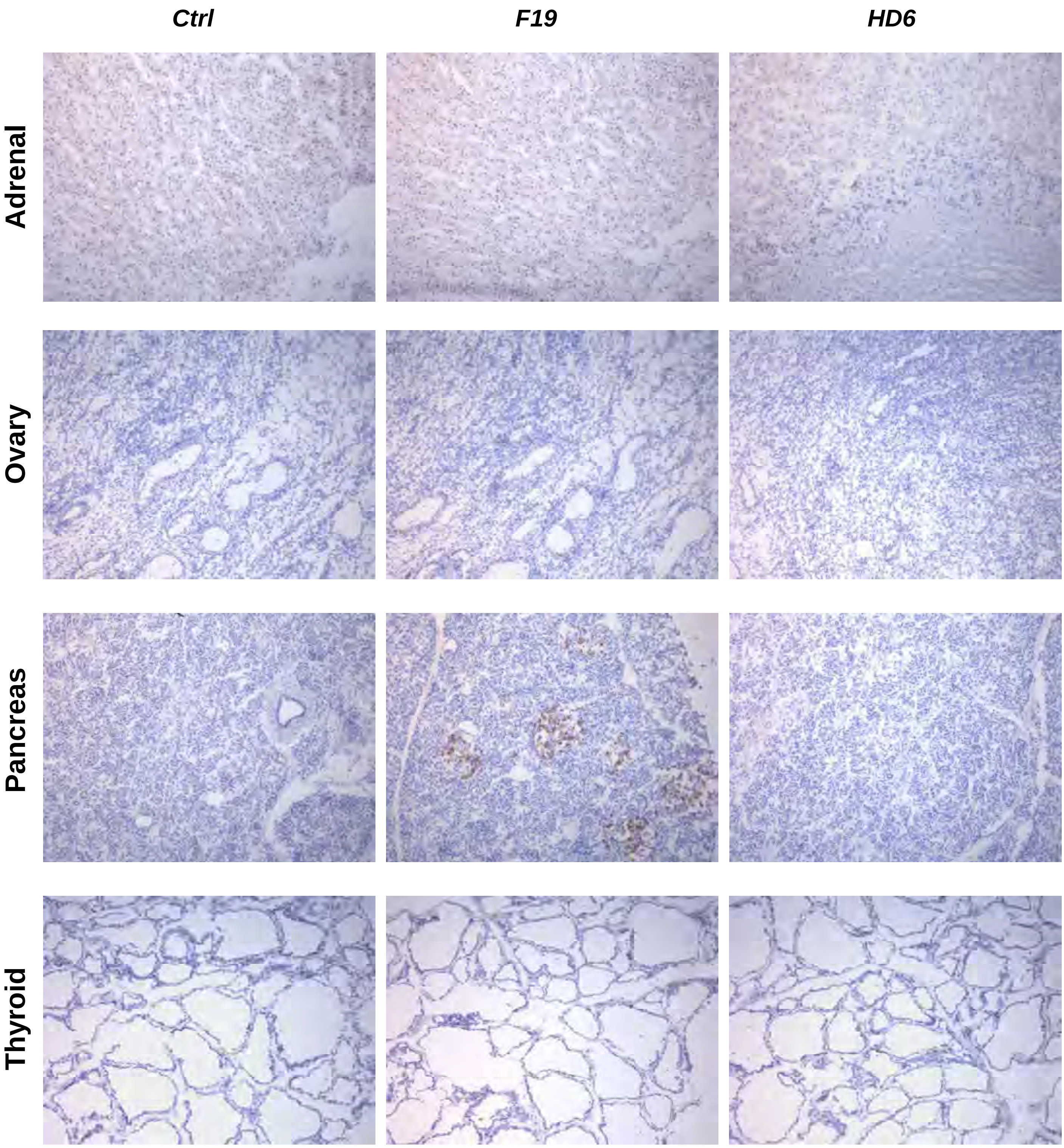

Figure S1: Immunohistochemistry staining for FAP of fresh-frozen human adult normal tissue. The left panels represent tissue staining with the secondary antibody only, the middle panels show tissue stained for FAP expression using the murine F19 antibody and the right panels have been stained with the control antibody murine HD6.

supplemental figure 1

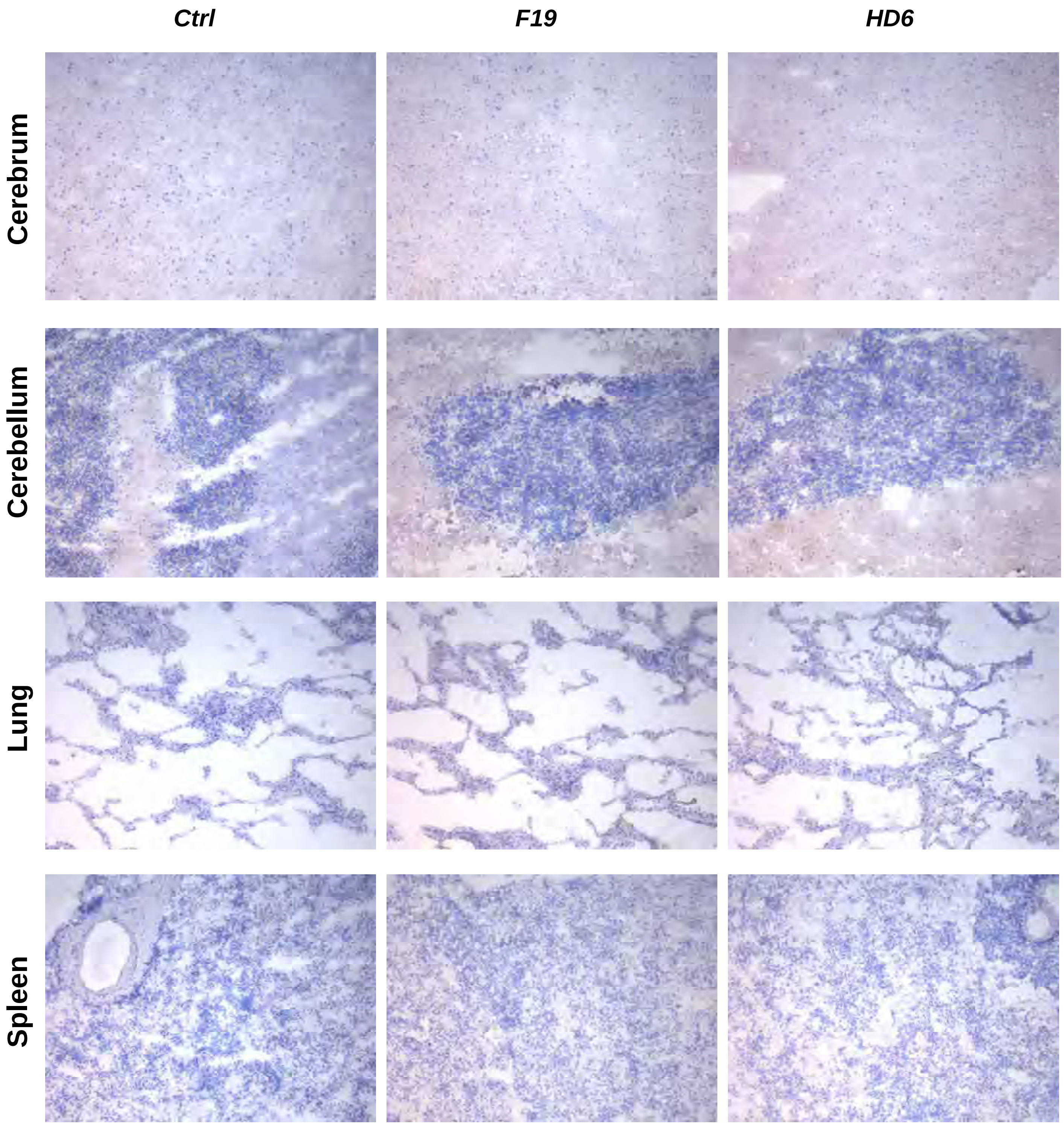

Figure S1: Immunohistochemistry staining for FAP of fresh-frozen human adult normal tissue. The left panels represent tissue staining with the secondary antibody only, the middle panels show tissue stained for FAP expression using the murine F19 antibody and the right panels have been stained with the control antibody murine HD6.

supplemental figure 1

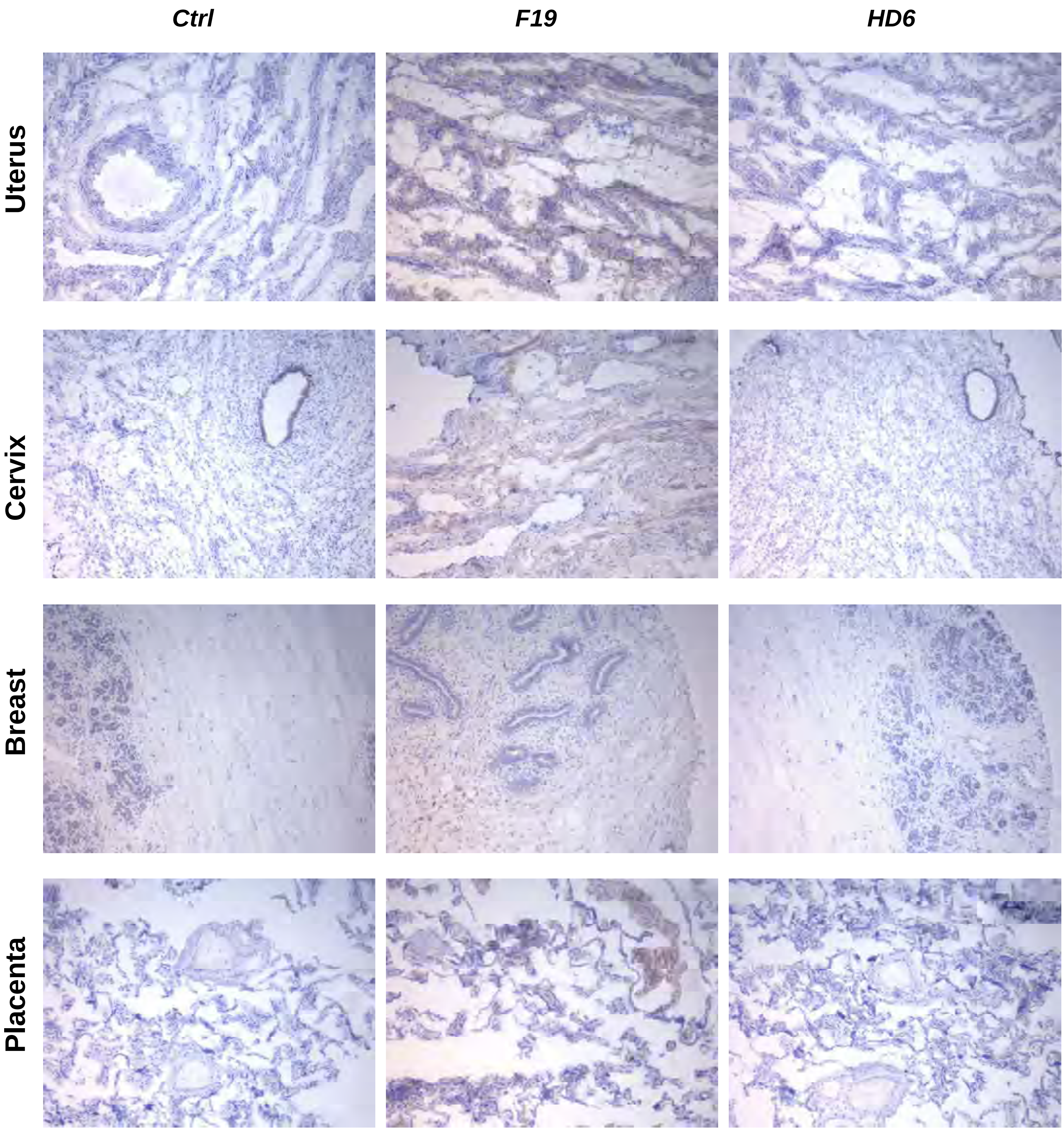

Figure S1: Immunohistochemistry staining for FAP of fresh-frozen human adult normal tissue. The left panels represent tissue staining with the secondary antibody only, the middle panels show tissue stained for FAP expression using the murine F19 antibody and the right panels have been stained with the control antibody murine HD6.

supplemental figure 1

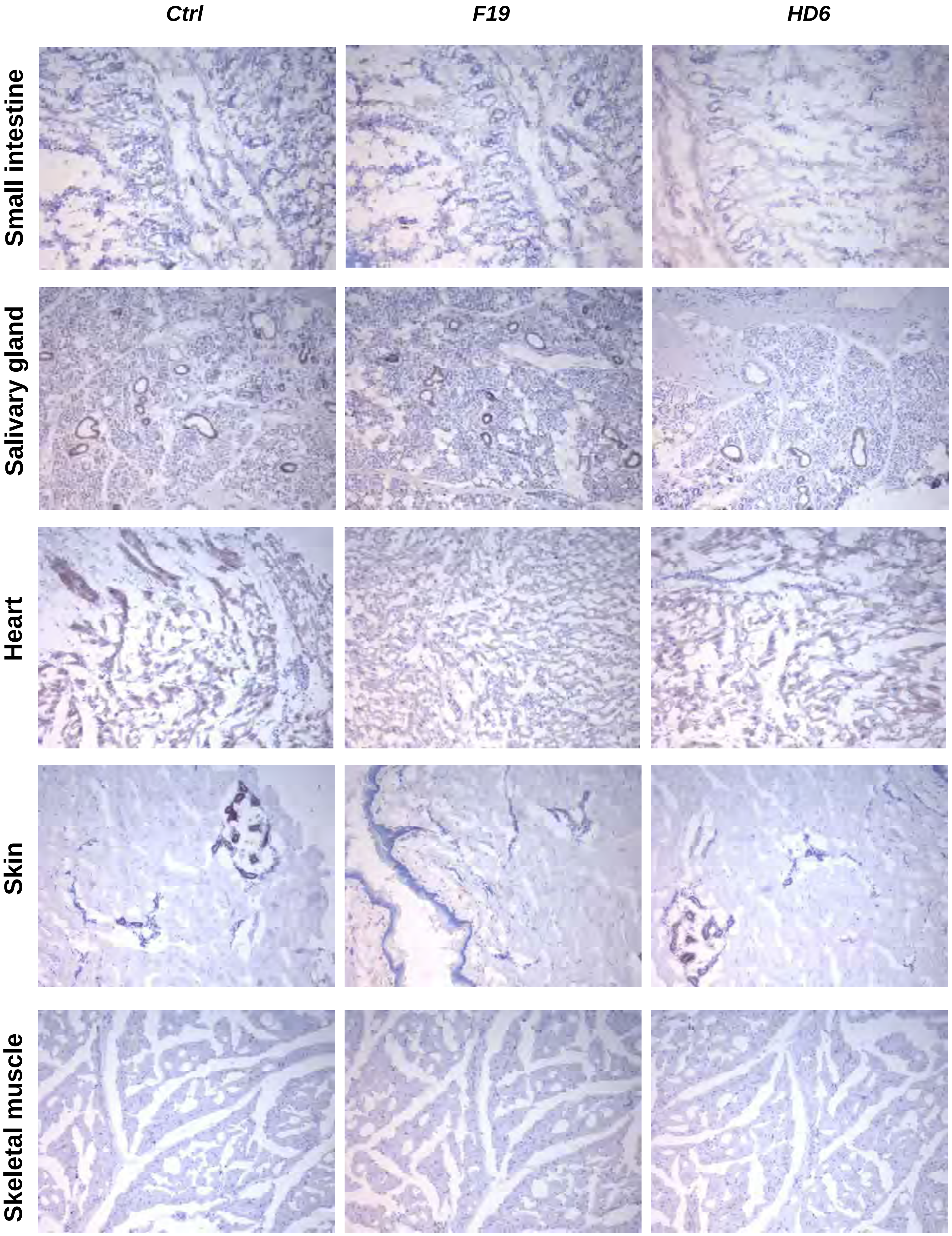

Figure S1: Immunohistochemistry staining for FAP of fresh-frozen human adult normal tissue. The left panels represent tissue staining with the secondary antibody only, the middle panels show tissue stained for FAP expression using the murine F19 antibody and the right panels have been stained with the control antibody murine HD6.

supplemental figure 1

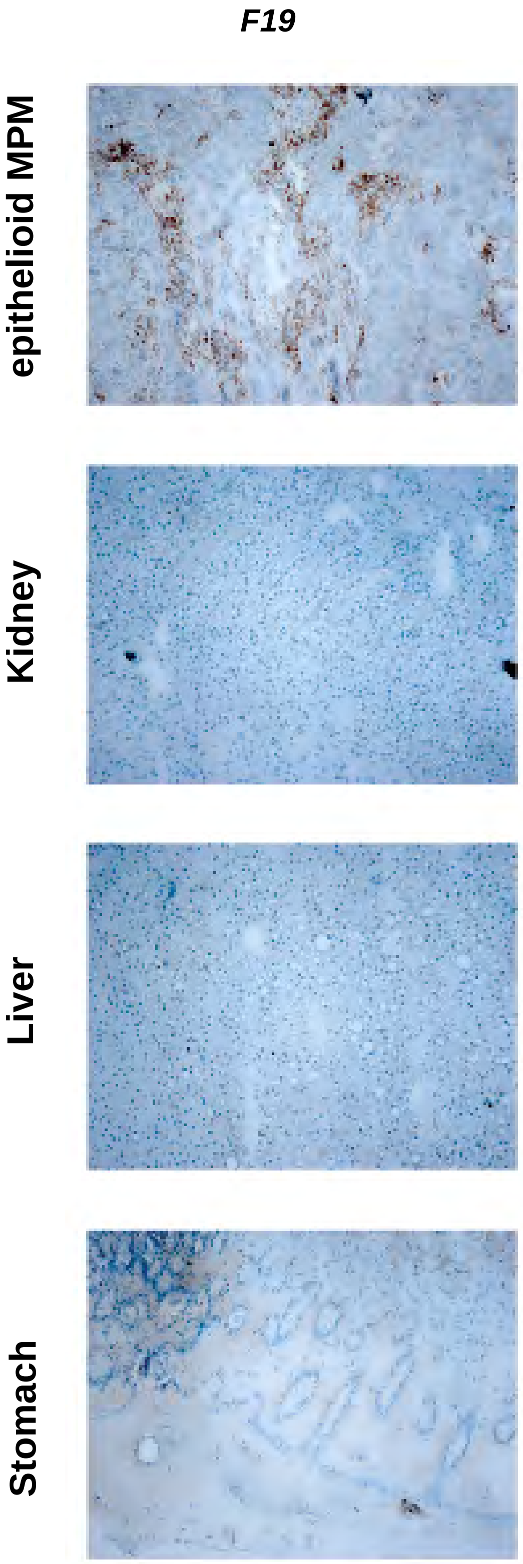

Figure S1: Immunohistochemistry staining for FAP of fresh-frozen human adult normal tissue using the murine F19 antibody. As specific staining control epithelioid malignant pleural mesothelioma (MPM) was used.

supplemental figure 2

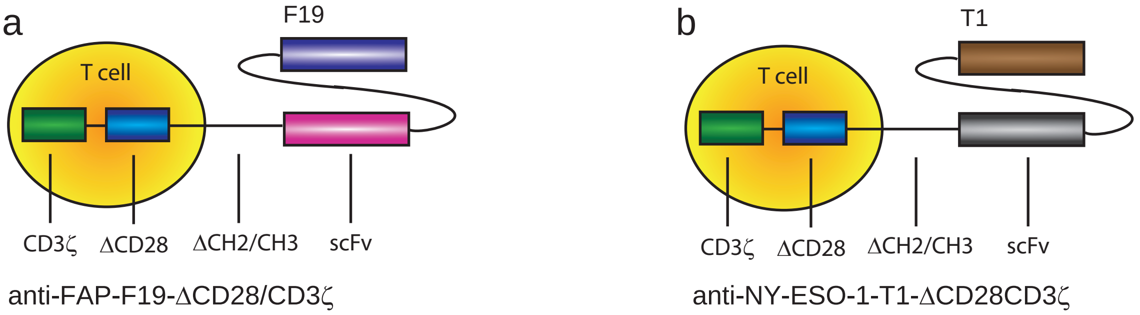

Figure S2: Schematic presentation of re-directed T cells harboring the different scFv. A displays re-directed T cells recognizing human FAP (anti-FAP-F19-ΔCD28/CD3ζ) and b re-directed T cells recognizing the NY-ESO-1$_{157\text{-}165}$ peptide complexed to HLA-A*02:01 (anti-NY-ESO-1-T1-ΔCD28/CD3ζ). Both scFv were cloned in the pBullet vector containing the human ΔCH2/CH3 domain, a ΔCD28 and a CD3ζ signaling domain.

supplemental figure 3

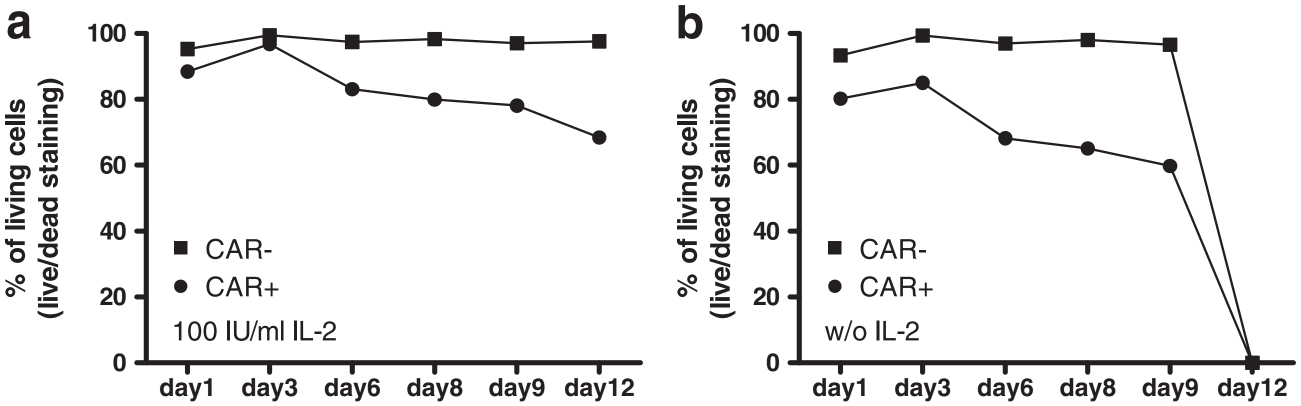

Figure S3: The polyclonal pool of anti-FAP-F19-ΔCD28/CD3ζ CAR transduced and non-transduced CD8+ T cells was cultured with (d) and without the addition of 100 IU/ml IL-2 (e). The percentage of living CD8+ T cells are shown for transduced (circles) and non-transduced (squares) T cells (n = 2).

supplemental figure 4

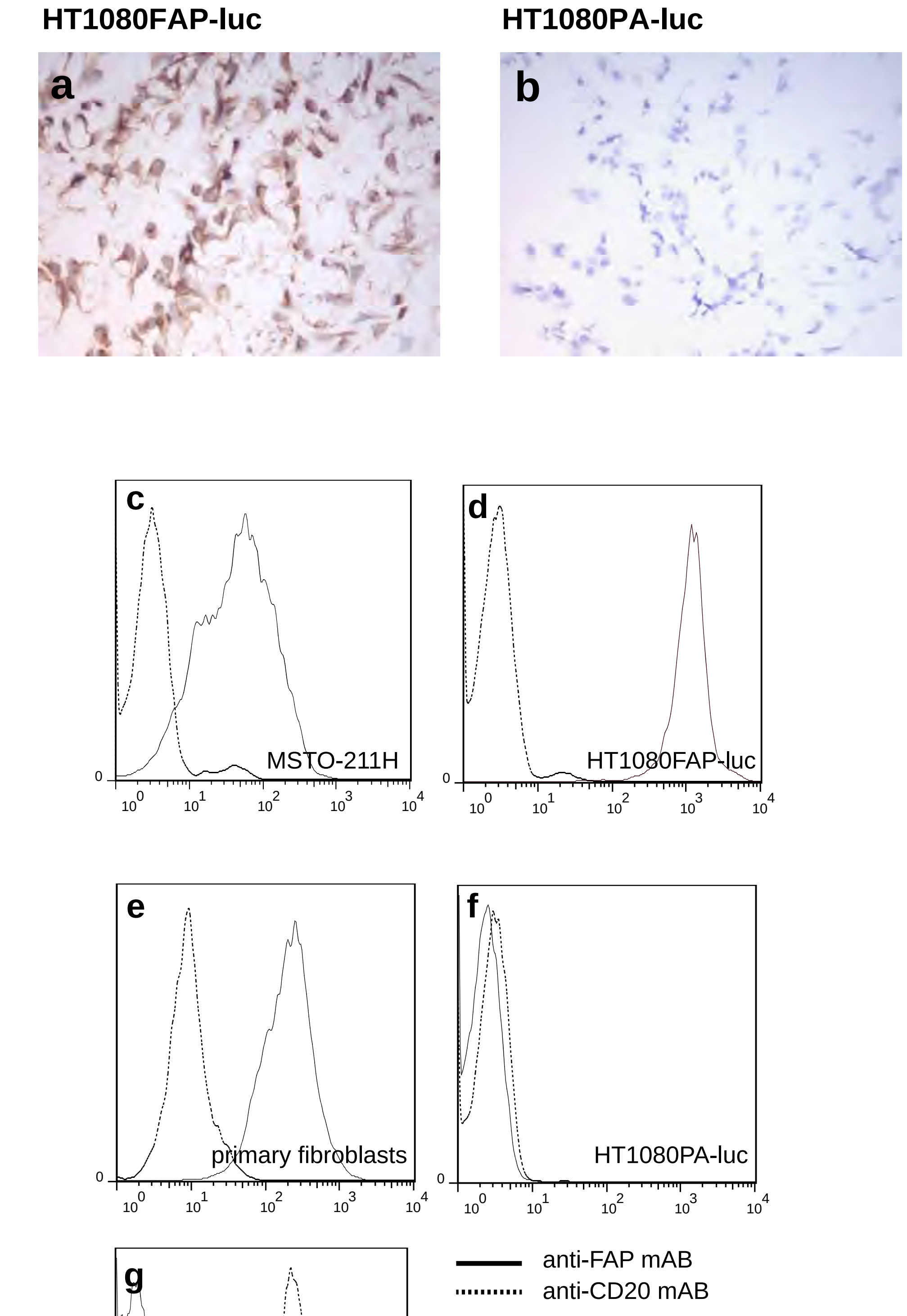

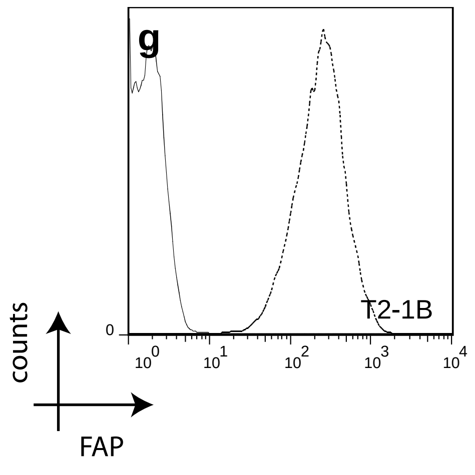

Figure S4: Immunohistochemistry staining for FAP of HT1080FAP-luc and HT1080PA-luc cell lines (a, b) and FAP expression levels on different target cell lines and primary fibroblasts (c-g). HT1080FAP-luc and HT1080PA-luc cells were stained with mF19 antibody to visualize FAP expression (a, b). Furthermore, MSTO-211H (c), HT1080FAP-luc (d), primary fibroblasts (e), HT1080PA-luc (f) and T2-1B (g) were analyzed for FAP expression levels by flow cytometry. The solid line represents staining of cells with the humanized F19 antibody, the dotted line shows staining with the control antibody (anti-CD20).
